# Mapping White Matter Microstructure in the One Month Human Brain

**DOI:** 10.1038/s41598-017-09915-6

**Published:** 2017-08-29

**Authors:** D. C. Dean, E. M. Planalp, W. Wooten, N. Adluru, S. R. Kecskemeti, C. Frye, C. K. Schmidt, N. L. Schmidt, M. A. Styner, H. H. Goldsmith, R. J. Davidson, A. L. Alexander

**Affiliations:** 1 Waisman Center, University of Wisconsin—Madison, Madison, WI USA; 2 Center for Healthy Minds, University of Wisconsin–Madison, Madison, WI USA; 3 Department of Psychology, University of Wisconsin—Madison, Madison, Wisconsin USA; 4 Department of Psychiatry, University of North Carolina–Chapel Hill, Chapel Hill, NC USA; 5 Department of Computer Science, University of North Carolina–Chapel Hill, Chapel Hill, NC USA; 6 Department of Psychiatry, University of Wisconsin—Madison School of Medicine and Public Health, Madison, WI USA; 7 Department of Medical Physics, University of Wisconsin—Madison School of Medicine and Public Health, Madison, WI USA

## Abstract

White matter microstructure, essential for efficient and coordinated transmission of neural communications, undergoes pronounced development during the first years of life, while deviations to this neurodevelopmental trajectory likely result in alterations of brain connectivity relevant to behavior. Hence, systematic evaluation of white matter microstructure in the normative brain is critical for a neuroscientific approach to both typical and atypical early behavioral development. However, few studies have examined the infant brain in detail, particularly in infants under 3 months of age. Here, we utilize quantitative techniques of diffusion tensor imaging and neurite orientation dispersion and density imaging to investigate neonatal white matter microstructure in 104 infants. An optimized multiple b-value diffusion protocol was developed to allow for successful acquisition during non-sedated sleep. Associations between white matter microstructure measures and gestation corrected age, regional asymmetries, infant sex, as well as newborn growth measures were assessed. Results highlight changes of white matter microstructure during the earliest periods of development and demonstrate differential timing of developing regions and regional asymmetries. Our results contribute to a growing body of research investigating the neurobiological changes associated with neurodevelopment and suggest that characteristics of white matter microstructure are already underway in the weeks immediately following birth.

## Introduction

The neural architecture that forms bundles of myelinated nerve fibers, known as white matter microstructure, is fundamental to brain connectivity and facilitates the advancement of higher-level cognitive functioning^1–4^. The development of white matter circuitry results from a cascade of intricate processes, such as axonal formation, dendritic sprouting, and myelination, that begins during the late stages of pregnancy and continues to develop through childhood, adolescence, and adulthood with the most pronounced maturation occurring within the first two years of life^5, 6^. During this developmental period, the neural substrates that govern individual differences toward vulnerability or resilience to adversity likely first begin to develop^7^. Despite the importance of white matter microstructure to healthy brain function and connectivity, a significant gap remains in our knowledge regarding normative characteristics of infant white matter microstructure, such as sexual dimorphism and asymmetry, particularly during the weeks immediately following birth.

Magnetic resonance imaging (MRI) provides detailed brain images and can non-invasively track changes associated with white matter development^8–10^. Diffusion MRI (dMRI) is highly sensitive to tissue microstructure and used extensively to study the fiber architecture of white matter. Diffusion tensor imaging (DTI) provides measurable metrics of diffusion characteristics, including fractional anisotropy (FA), mean diffusivity (MD), radial diffusivity (RD), and axial diffusivity (AD), each of which provides quantitative characteristics for the underlying diffusivity of the brain. Given the degree of anisotropic diffusion in white matter – due to tissue barriers such as axonal fibers and the myelin sheath – FA, MD, RD, and AD provide indirect markers of white matter microstructure^11, 12^.

More recent dMRI techniques, such as neurite orientation dispersion and density imaging (NODDI^13^), use biophysical modeling to improve the level of microstructure specificity available from neuroimaging. NODDI measures quantitative parameters of three specific diffusion processes: intraneurite diffusion (ν_IC;_ diffusion within axons and dendrites), extraneurite diffusion, and isotropic (free) water diffusion (ν_ISO_). The model also quantifies the degree of angular variation of the neurites through the orientation dispersion index (ODI). NODDI provides important information regarding brain development in infants and young children, with parameter changes consistent with neurodevelopmental mechanisms of myelination and axonal fiber development during the first few years of life^14–18^. For instance, nonlinear increases in the NODDI intraneurite volume fraction (ν_IC_) occur across approximately the first 3 years of childhood^17^, and ν_IC_ continues to increase nonlinearly up to approximately 7.5 years of age^18^. In very preterm children, NODDI parameters indicate increased axon dispersion and lower axon density compared to control infants, and these parameters also associated with poorer functional outcomes in the very preterm children^14^. These studies provide valuable insight into developmental profiles of the brain while also demonstrating the utility of dMRI methodology.

While diffusion MRI has greatly contributed to our understanding of white matter development and microstructural changes that occur throughout early development^10^, few studies have examined the normative white matter characteristics immediately following birth^19, 20^. Studies have compared DTI and NODDI between full-term and preterm infants^14, 15, 21, 22^, while others have examined these measures across older infants and children^23–25^. However, research has yet to systematically study the regional variation of DTI and NODDI microstructural measures in a large sample of infants during the early postnatal period. A better understanding of the regional variations and characteristics in the normative neonate brain are necessary to establish a baseline that is not yet influenced by postnatal experiences. In doing so, researchers will be better able to examine the sensitivity and susceptibility of white matter microstructure to subsequent life experiences and exposures. Further, though we know that sexual dimorphisms and regional asymmetries exist in adolescent and adult white matter^26, 27^, we do not know whether such features are present at very early stages of life or if these develop over time.

In this work, we extend recent work investigating infant white matter development and investigate the regional variations and characteristics of microstructure that are present during the neonatal period. An optimized acquisition protocol was developed to acquire multiple b-value diffusion weighted images from 1 month old infants during natural, non-sedated sleep. We quantify DTI and NODDI parameters within white matter regions and assess associations of these measures with gestation corrected age, regional asymmetries, infant sex, and markers of child development, such as head circumference and birth weight and length. Finally, we compare white matter microstructure measures between DTI and NODDI at this early stage of development to assess whether these measures are sensitive to similar or dissimilar aspects of the microstructure. These analyses provide an important step for understanding the normative features of white matter microstructure present at the first month of life.

## Methods

### Participants

Pregnant women were recruited as part of an ongoing longitudinal study examining the influence of early life experiences on brain and behavioral development in infants. Participating women were identified during the second trimester of pregnancy (<28 weeks’ gestation) and considered for inclusion based on the following criteria: between 18 and 40 years of age, expecting singleton births, no diagnosis of psychotic illnesses (i.e., schizophrenia, bipolar disorder, borderline personality disorder), no pre-existing neurological conditions or major head trauma, no major autoimmune disease or infections during pregnancy, and uncomplicated childbirth. Families were additionally excluded for any exposure to the neonatal intensive care unit (NICU) and if the infant did not go home with the mother at discharge. These criteria were confirmed by mothers through interviews prior to enrollment and through medical history questionnaires obtained during the study.

The University of Wisconsin–Madison Institutional Review Board approved all study procedures, and written informed consent was obtained from the parents of each participating family. All experiments were performed in accordance with relevant guidelines and regulations.

### Data Acquisition

Infants underwent imaging at 1 month of age during natural, non-sedated sleep^28^; MRI visits were scheduled to correspond with the infant’s daily nap schedule and typically occurred after the infant was fed and swaddled. Once asleep, the infants were moved into the darkened MRI scanner suite, where several techniques to enhance imaging data collection in sleeping infants were implemented. For instance, infants were swaddled with an infant MedVac vacuum immobilization bag (CFI Medical Solutions, USA) and foam cushions were placed around their heads to reduce intra-scan motion. We reduced the acoustic noise experienced by the sleeping infant by fitting a foam insert to the inside of the scanner bore, utilizing both malleable ear plugs and MiniMuff®(Natus Medical Incorporated) neonatal noise-attenuating ear covers, and using electrodynamic headphones (MR Confon, Germany) that played white noise during the image acquisition.

Imaging data were acquired using a 3 Tesla General Electric MR750 Discovery scanner equipped with a 32 channel receive-only head RF array coil (Nova Medical, Wakefield, MA). Optimized infant imaging protocols were developed to further reduce the acoustic noise of the scanner and ensure that infants remained asleep throughout the scan by limiting the peak gradient amplitudes and slew-rates of pulse sequences. Infants were continuously monitored throughout the MRI scan by a trained research staff member, and mothers remained in the scanner room throughout the scan if they chose to do so.

A three-shell diffusion weighted imaging (DWI) protocol was acquired using a single shot spin-echo echo-planar imaging pulse sequence. Parallel acquisition with a geometric reduction factor of two was used to reduce image acquisition time and distortions from magnetic field inhomogeneities. A total of 69 DWIs were acquired, 6 of which were acquired with no diffusion encoding (i.e., b-value = 0 s/mm^2^). The remaining 63 images were acquired along non-collinear diffusing encoding directions with b = 350 s/mm^2^ [9 directions], b = 800 s/mm^2^ [18 directions], and b = 1500 s/mm^2^ [36 directions]. Imaging parameters consisted of the following: repetition time [TR] = 8400 ms, echo time [TE] = 94 ms, and bandwidth = 3906 Hz/pixel. Imaging field of view [FOV] was 25.6 cm × 25.6 cm with an acquisition matrix of 128 × 128, providing a 2mm × 2mm in-plane resolution. Coverage across the cerebrum and cerebellum was achieved by acquiring 60 sagittal-oriented contiguous slices with a slice thickness of 2.0 mm. The total time for the multiple b-value DTI acquisition using strategies to reduce the acoustic noise was approximately 10 minutes.

### Image Preprocessing

Following image acquisition, DWIs were manually assessed for motion artifacts, and individual encoding direction volumes were subsequently dropped if artifacts (e.g., signal dropout), were present. Distortion, translation and rotation from bulk head motion and eddy currents were accounted for by co-registering DWIs using an affine registration tool^29^ from the FMRIB software library (FSL; http://fsl.fmrib.ox.ac.uk/fsl/fslwiki/) software suite. Gradient directions were additionally corrected for rotations^30^. Non-parenchyma signals were removed using the 3dSkullStrip tool as part of the Analysis of Functional NeuroImages (AFNI) software package (http://afni.nimh.nih.gov/pub/dist/doc/program_help/3dSkullStrip.html) and diffusion tensors were estimated for each voxel using the robust estimation of tensors by outlier rejection (RESTORE^31^) algorithm as part of the diffusion imaging in python (DIPY) open source software package^32^. As the largest b-value of the acquisition consisted of 1500 s/mm^2^, all three shells were utilized in fitting the diffusion tensors. Eigenvalues (λ_1_, λ_2_, λ_3_) were calculated from these voxel-wise estimates of the diffusion tensor, and quantitative maps of FA, MD, RD, and AD, were derived^33^.

Pre-processed diffusion data were next fit to the three-compartment NODDI tissue model to provide neurite density and dispersion estimates^13^. This tissue model was fit using the available MATLAB toolbox (nitrc.org) and adapting it to run on the condor parallel computing environment (https://github.com/nadluru/NeuroImgMatlabCondor). Default model assumptions and fixed parameter values as described in Zhang *et al*. (2012) were used in the fitting of the NODDI model. From this model fit, the tissue model parameters of ν_IC_, ν_ISO_, and ODI are estimated. Within the NODDI formulation, restricted diffusion is attributed to axons and dendrites (neurites), and thus ν_IC_ is interpreted as a quantitative measure of neurite density, while the volume fraction of the isotropic diffusion compartment, ν_ISO_, is attributed to cerebrospinal fluid (CSF) or isotropic diffusion. ODI is a parameter that quantifies the angular variation of the neurite orientation (i.e., dispersion/fanning) and is thus sensitive to the degree of fiber coherence^13, 34^.

**Figure 1:**
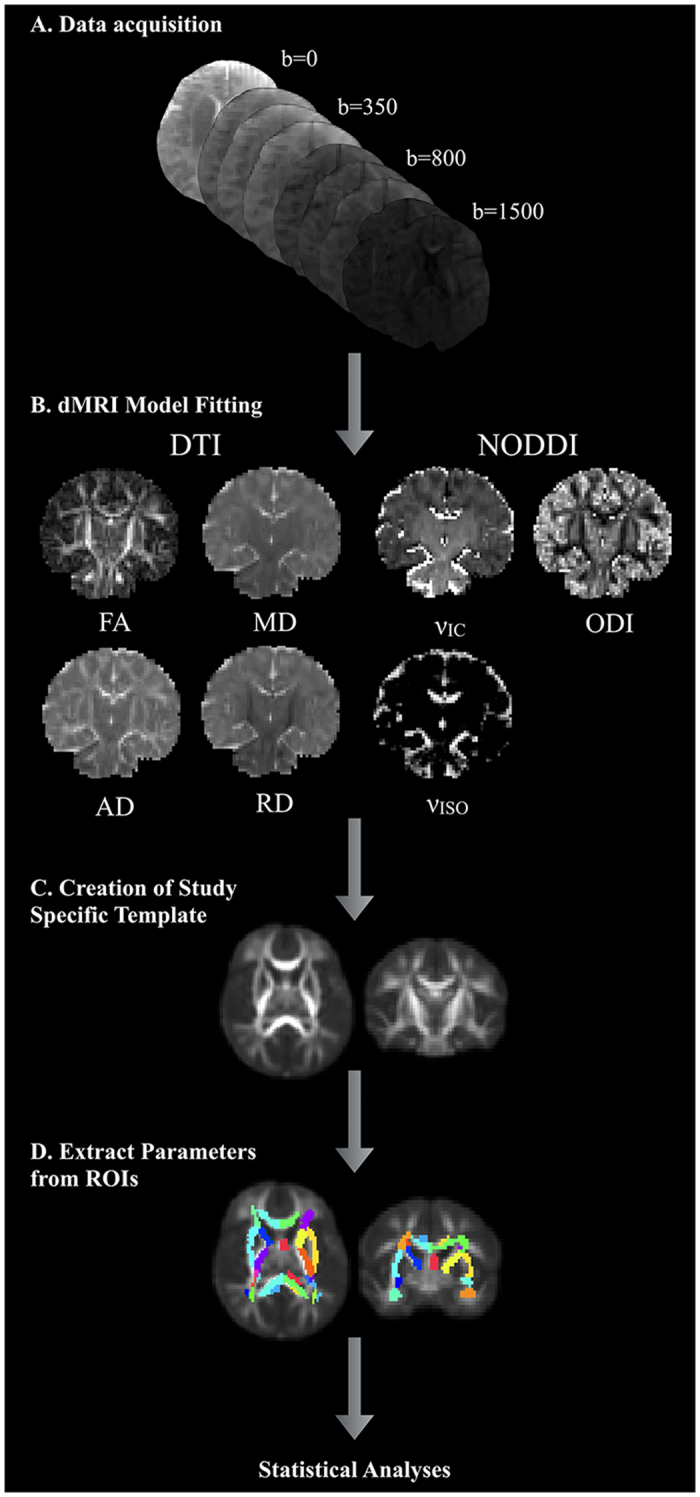
Overall schematic depicting the acquisition, processing, and analyses of the current study.

DTI-TK was used to generate a population-specific template using affine and diffeomorphic diffusion tensor registration^35^ using the full diffusion tensors from a representative subset of the study cohort. Next, each subject’s FA map was nonlinearly aligned to the FA map of the study-specific template using Advanced Normalization Tools (ANTs) diffeomorphic registration algorithms^36–38^. White matter regions as provided in the Johns Hopkins University neonatal atlas^39^ were spatially aligned to the study-specific template again using ANTs and nearest neighbor interpolation. The normalized neonatal atlas was then warped into each subject’s native space by applying the inverse of the spatial transformations estimated from the registration of the subject data to the template. Native-space FA, MD, RD, AD and ν_IC_, ν_ISO_, and ODI maps were subsequently parceled into the 122 available white matter tracts and brain regions contained within the atlas. Median values for the DTI and NODDI parameters were computed from a subset of the available white matter regions and participant, as the median is less sensitive to voxels with extreme values compared to the mean^40^. An overall schematic of the DTI processing is depicted in Fig. 1.

White matter regions throughout the brain were selected from the atlas for analysis, including the following: corpus callosum; anterior limb, posterior limb and retrolenticular part of the internal capsule; anterior, superior and posterior corona radiate; the cingular and hippocampal part of the cingulum; stria terminalis; superior longitudinal fasciculus; external capsule; posterior thalamic radiation; sagittal stratum; superior and inferior fronts-occipital fasciculus; and the uncinate fasciculus (Supplementary Fig. 1).

### Statistical Analyses

#### Relations with Gestation Corrected Age

Infants were scanned at 1 month of age ±2 weeks depending on scheduling, resulting in variation in gestation corrected age at the time of the scan. We used linear models to examine age-related changes in the DTI and NODDI parameters. All infant postnatal ages were corrected to a 40-week gestation and significance was defined as α < 0.0015 (*p* < 0.05, Bonferroni corrected for the 34 white matter regions examined).

#### Regional Asymmetry and Sex Differences

We examined differences between left and right homologous white matter regions using paired t-tests, and differences between males and females were evaluated using independent t-tests. To account for possible gestational age differences between males and females, we also compared DTI and NODDI measures between males and females after controlling for gestation corrected age. Linear regression was used to regress the effect of gestation corrected age and independent t-tests compared the residualized DTI and NODDI measures between males and females. We again used Bonferroni correction to account for multiple comparisons and significance was defined as *p* < 0.05, corrected for multiple comparisons.

#### Relations with Markers of Newborn Growth

Participant medical records were used to assess growth measures of newborn head circumference, birth weight, and birth length. Partial Pearson correlations (Bonferroni corrected with significance defined as *p* < 0.05) relate newborn growth measures and the dMRI (DTI and NODDI) parameters for each white matter region, controlling for gestation corrected age.

#### Comparison of DTI and NODDI

Associations between DTI (FA, MD, RD, and AD) and NODDI (ν_IC_, ν_ISO_, and ODI) measurements were examined using partial Pearson product-moment correlations calculated for each white matter region, controlling for gestation corrected age. Statistical significance was defined at p < 0.05 (Bonferroni corrected for multiple comparisons).

## Results

A total of 149 mothers and their infants participated in the MRI scanning procedures. Of the total sample, 33 infants woke up prior to and 13 infants woke up during the diffusion acquisition, resulting in a final study sample of 104 (50 male), with a mean age of 32.7 days ± 5.81 days (corrected to a 40-week gestational period). Males and females were similar, with no significant mean differences on gestation corrected age at MRI scan (*p* = 0.58), gestation length (*p* = 0.69), head circumference at birth (*p* = 0.32), birth weight (*p* = 0.98), birth length (*p* = 0.52), or maternal age at birth (*p* = 0.66). Additional summary and demographic statistics are in Table 1.

**Table 1:** Demographic information for study cohort.

Sample Characteristics	Males	Females	Combined	P-Value
N	50	54	104	
Mean Age at MRI (days, corrected to 40 week gestation)	32.4 (5.5)	33.0 (6.1)	32.7 (5.8)	0.59
Mean Gestational Age at birth (days)	−3.54 (9.7)	−2.78 (9.7)	−3.14 (9.6)	0.69
Mean Gestation Length (weeks)	39.5 (1.4)	39.6 (1.4)	39.6 (1.4)	0.69
Mean Birth weight (kg)	3.49 (0.56)	3.48 (0.50)	3.48 (0.52)	0.90
Mean Birth Length (cm)	20.0 (2.0)	20.2 (1.1)	20.1 (1.6)	0.52
Mean Head Circumference (cm)	35.0 (1.5)	34.5 (1.4)	34.7 (1.4)	0.08
**APGAR Score**				
Median 1 Min (Range)	8 (1–9)	9 (1–9)	8.5 (1–9)	0.60
Median 5 Min (Range)	9 (5–10)	9 (6–9)	9 (5–10)	0.75
Mean Maternal Age (years)	32.3 (4.2)	32.0 (3.5)	32.2 (3.8)	0.66
**Racial Background**				
African American/Black	0	1	1	
Asian	3	1	4	
Caucasian/White	44	44	88	
Native Hawaiian or Other Pacific Islander	0	0	0	
Mixed Race	1	4	5	
Not Reported	2	4	6	

**Figure 2:**
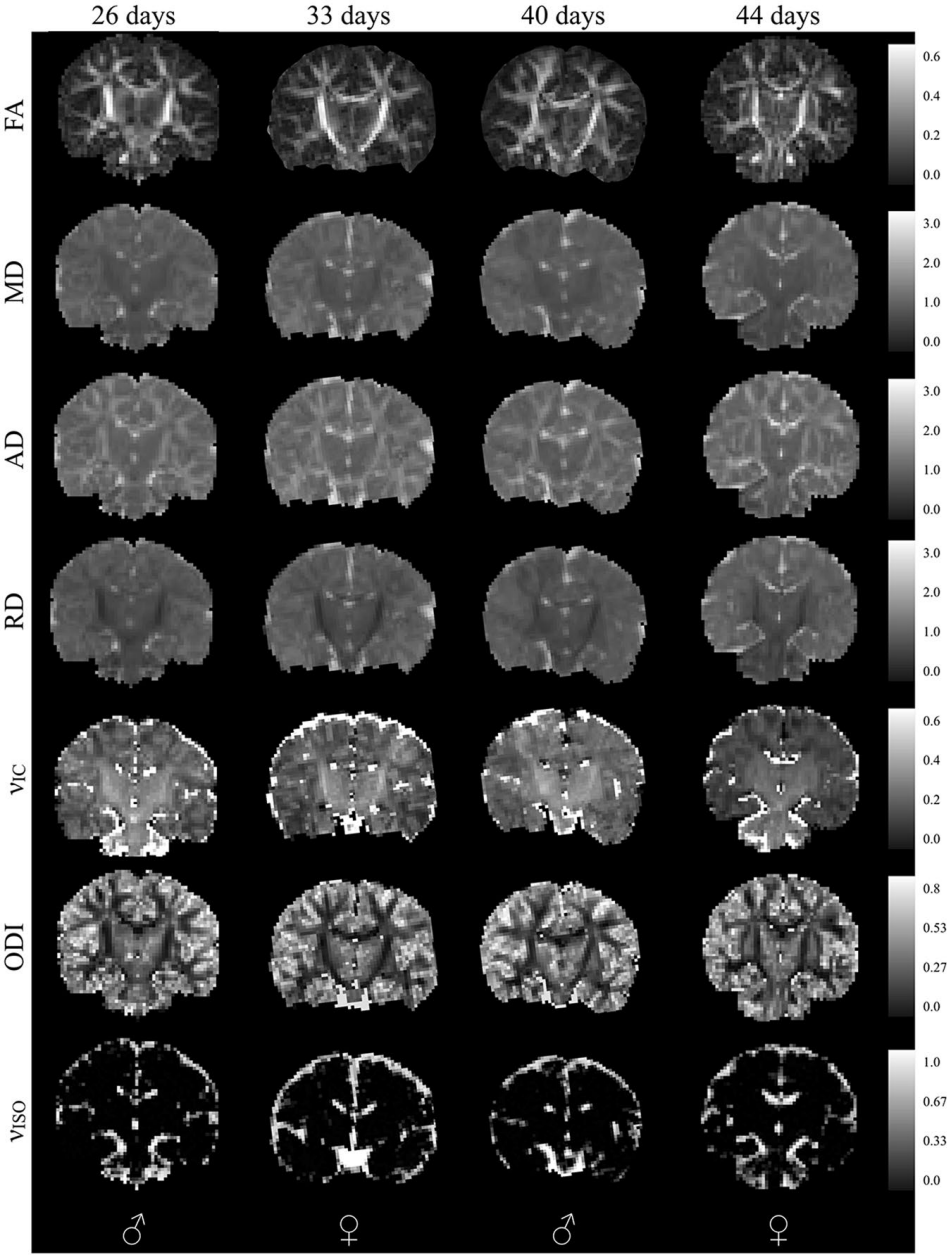
Coronal raw DTI (FA, MD, AD, and RD) and NODDI (ν_IC_, ODI, and ν_ISO_) estimates from four representative infants. The sex of the infant is denoted in the bottom panel. The optimized diffusion protocol resulted in high quality DWI data and parameter maps.

**Figure 3:**
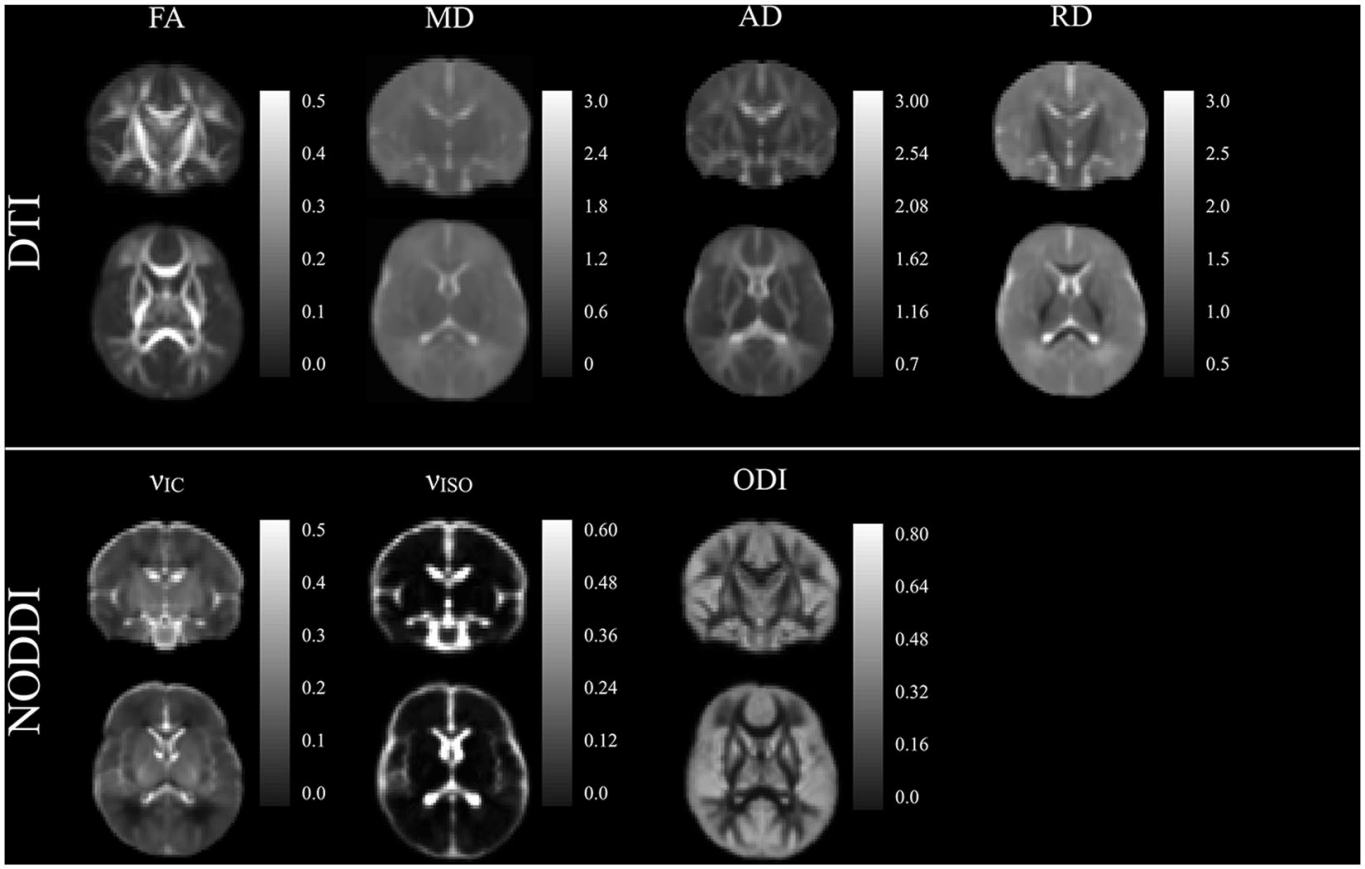
Matched coronal and axial slices through the of the population averaged DTI and NODDI parameters.

DTI and NODDI models were estimated for each of the 104 infants. On average, 13.88% (between 9–10) of DWIs were dropped from each participant during the quality control procedure due to motion related artifacts, with most of these images being from the largest b-value. We assessed DTI and NODDI maps following model fitting to ensure the parameter maps were of high quality. Coronal sections of raw DTI and NODDI parameter maps from four representative infants are shown in Fig. 2, while corresponding matched axial and coronal images of the mean DTI and NODDI parameter estimates are in Fig. 3. Overall, these individual maps demonstrate the ability to acquire high quality multiple b-value dMRI and successfully estimate DTI and NODDI based parameters during this early developmental period.

### White Matter Neurodevelopment

Qualitatively, parameter maps depict early developing white matter microstructure that is consistent with neurodevelopmental patterns in the newborn. MD is relatively diffuse across the gray and white matter. FA, AD, and RD provide increased differentiation between gray and white matter microstructure, with FA and AD having higher values within white matter. RD, on the other hand, has the smallest values in the corpus callosum and internal capsules, likely reflecting the earliest myelination of these structures^18, 41, 42^. ODI has the smallest values within the corpus callosum and internal capsules, reflecting the early organization and low dispersion of these tracts, while tracts further out from the center of the brain have increased ODI values that are indicative of less mature white matter. Increased values are observed in medial white matter, such as the brain stem and internal capsules, whereas values decrease laterally in the superficial white matter.

### Gestation Corrected Age

**Figure 4:**
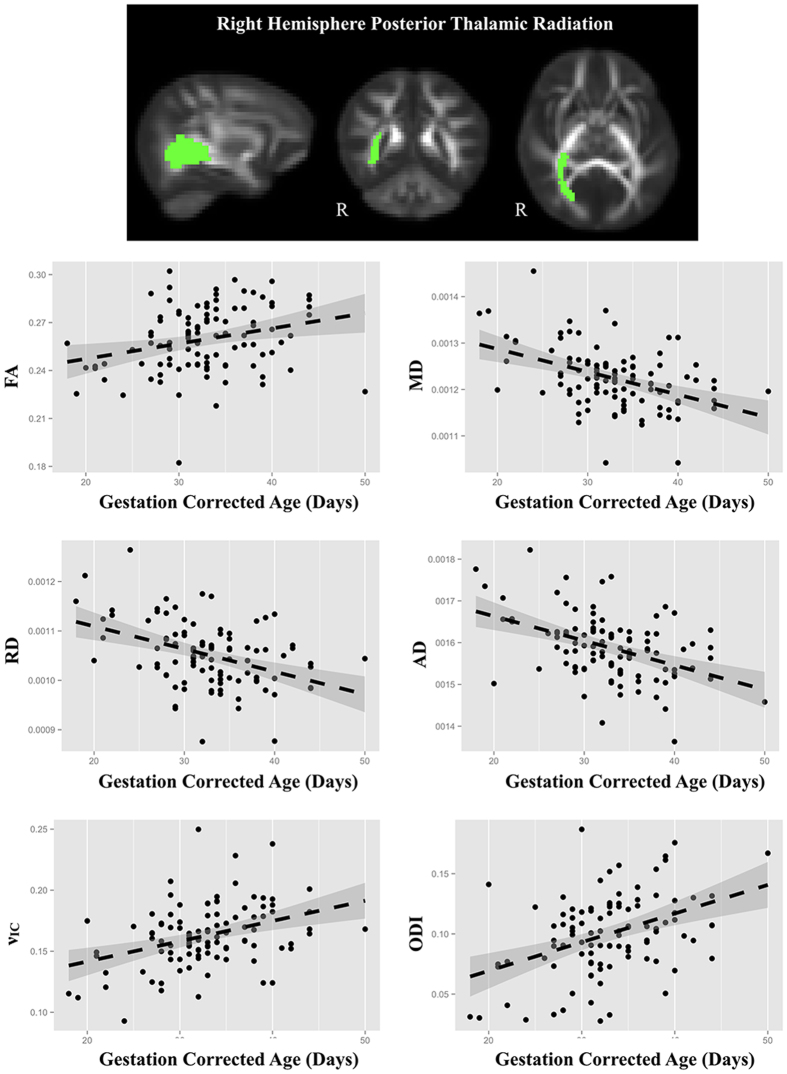
Representative scatter plots of the gestation corrected age relationships observed for the posterior thalamic radiations. Points correspond to the mean values from the posterior thalamic radiations. In general, increases of FA, ν_IC_, and ODI and decreases of diffusivity measures were observed across the majority of white matter regions.

Infants were approximately 1 month old, but there was variability in gestational corrected age that related to regional measures of white matter microstructure. Many white matter regions, including the internal capsules, corona radiata, and superior longitudinal fasciculus, varied with gestation corrected age (Table 2). Representative scatter plots of age relations for the right-hemisphere posterior thalamic radiation are shown in Fig. 4. In general, FA increased and MD, AD, and RD decreased, consistent with patterns of developing microstructure. ν_IC_ increased across many of the same white matter regions, suggesting that developing neurite microstructure may be responsible for the DTI parameters’ changing patterns. ODI was increased within the right-hemisphere superior corona radiate and left-hemisphere cingulum and posterior thalamic radiation. ν_ISO_ did not vary with gestation corrected age.

**Table 2:** Summary of associations (T-statistics) of DTI and NODDI parameters with gestation corrected age.

Left Hemisphere	DTI				NODDI		
	FA	MD	AD	RD	ν_IC_	ODI	ν_ISO_
Corpus Callosum	0.40	−2.97*	−2.94*	−1.83	1.67	2.55	2.50
Anterior Limb of Internal Capsule	0.60	−2.22	−2.01	−2.06	1.94	1.31	0.46
Posterior Limb of Internal Capsule	0.74	−**4.49**	−3.23*	−**3.46**	**3.70**	2.33	0.91
Retrolenticular Part of Internal Capsule	0.96	−2.09	−1.25	−2.27	1.10	0.22	−0.24
Anterior Corona Radiata	**4.88**	−**3.70**	−3.00*	−**4.09**	2.08	0.56	0.86
Superior Corona Radiata	2.15	−**4.17**	−**3.97**	−**4.03**	**3.98**	**3.64**	0.88
Posterior Corona Radiata	1.67	−**4.19**	−**3.32**	−**4.53**	**3.28**	2.41	0.43
Cingulum (cingular part)	0.21	−**4.87**	−**4.52**	−**4.53**	**3.89**	2.49	1.18
Cingulum (hippocampal part)	−0.28	−1.22	−0.57	−0.63	1.09	0.20	1.55
Stria Terminalis	1.35	−**3.40**	−3.06*	−**3.51**	2.93*	1.33	−0.67
Superior Longitudinal Fasciculus	**4.72**	−**5.09**	−**4.40**	−**5.33**	**4.30**	2.46	−0.48
External Capsule	3.26*	−**4.34**	−**3.44**	−**5.00**	**3.32**	1.29	0.91
Posterior Thalamic Radiation	1.91	−2.55	−1.77	−2.42	1.83	1.49	1.39
Sagittal Stratum	2.31	**−3.39**	−2.17	−**3.32**	3.17*	1.66	2.38
Superior Fronto−Occipital Fasciculus	0.91	−1.64	−1.33	−1.78	1.74	1.44	2.60
Inferior Fronto−Occipital Fasciculus	0.88	−**3.99**	−2.81*	−**3.56**	2.76*	1.72	0.77
Uncinate Fasciculus	1.24	0.81	1.42	0.33	−1.60	−1.90	−1.54
**Right Hemisphere**							
Corpus Callosum	0.46	−0.96	−0.53	−1.20	1.34	1.84	2.40
Anterior Limb of Internal Capsule	2.95*	−**4.18**	−3.00*	−**4.53**	**3.81**	0.88	0.51
Posterior Limb of Internal Capsule	2.55	−3.24*	−1.65	−3.23*	2.28	−0.53	−0.39
Retrolenticular Part of Internal Capsule	−0.01	−2.95*	**−3.85**	−2.84*	2.74*	3.13*	1.02
Anterior Corona Radiata	**4.51**	−**3.71**	−2.60	−**3.84**	2.81*	1.05	0.57
Superior Corona Radiata	2.09	−**4.04**	−**3.75**	−**3.84**	**3.59**	2.32	−0.22
Posterior Corona Radiata	1.40	−**3.45**	−3.07*	−**3.35**	2.83*	1.43	−0.56
Cingulum (cingular part)	0.92	−**3.32**	−**3.99**	−2.40	2.71*	**3.45**	1.10
Cingulum (hippocampal part)	−0.26	−2.98*	−**3.60**	−1.76	1.44	2.03	1.22
Stria Terminalis	1.59	−2.23	−1.83	−2.34	1.84	1.27	−0.11
Superior Longitudinal Fasciculus	1.79	−**4.37**	−**4.17**	−**4.34**	**3.93**	2.34	1.73
External Capsule	0.85	−**3.81**	−**4.46**	−3.21*	2.90*	2.33	0.89
Posterior Thalamic Radiation	2.83*	−**4.84**	−**4.93**	−**4.56**	**4.08**	**4.50**	−1.63
Sagittal Stratum	3.24*	−**4.69**	−**3.99**	−**4.72**	**4.17**	3.00*	−1.61
Superior Fronto−Occipital Fasciculus	1.50	−3.24*	−2.45	−2.94*	2.48	1.07	−0.21
Inferior Fronto−Occipital Fasciculus	2.04	−3.10*	−2.71*	−3.17*	2.22	−0.05	0.23
Uncinate Fasciculus	1.94	−0.95	−0.08	−1.46	0.08	−0.67	0.49

Significant (p < 0.05, Bonferroni corrected) associations are denoted by bold type. Trending (p < 0.01, uncorrected) associations are denoted by *.

**Figure 5:**
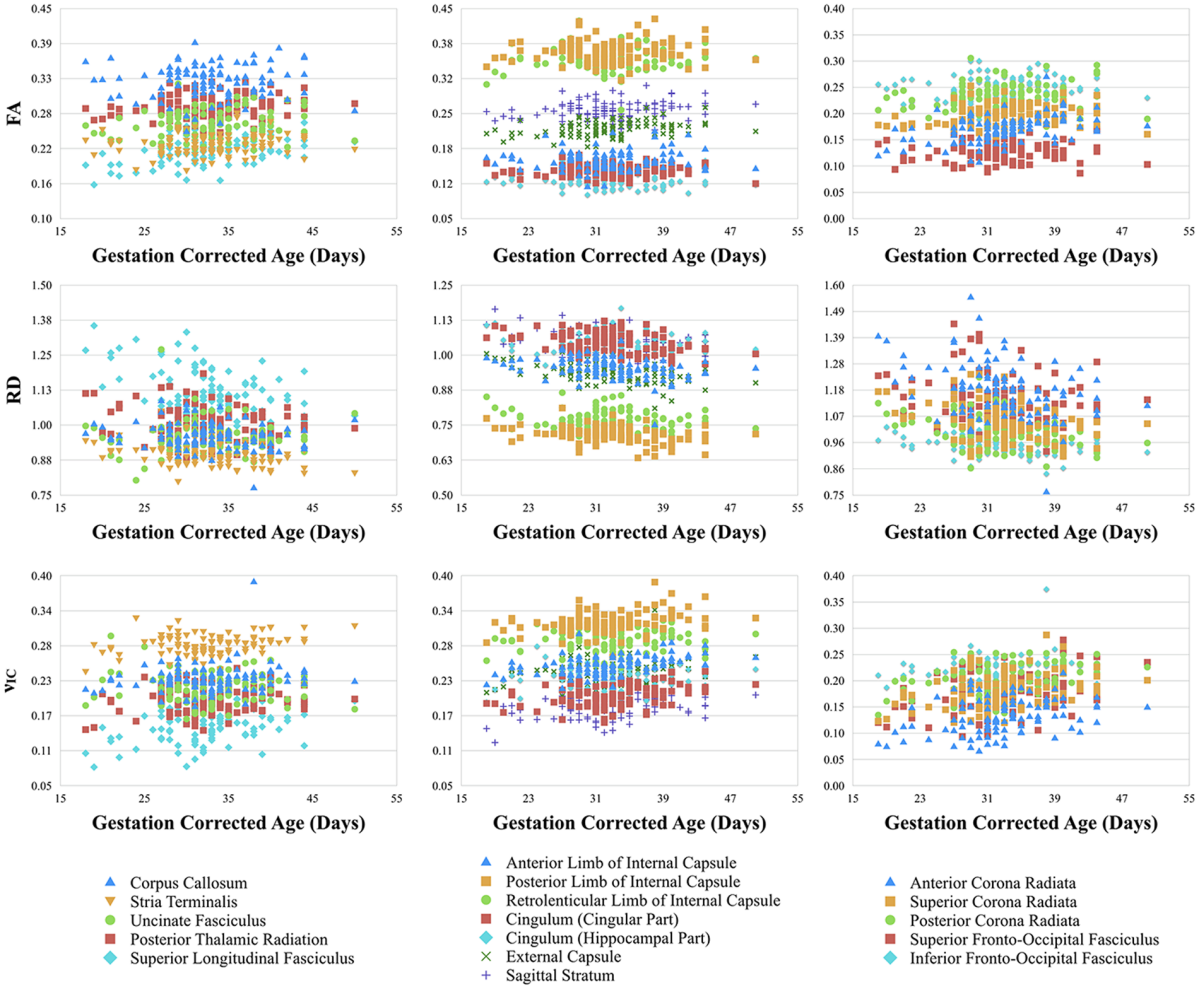
Combined gestation corrected age relationships of FA, RD and ν_IC_ for white matter regions of the left hemisphere. The legend under each column indicates the depicted white matter regions.

In addition to observing significant age relations of DTI and NODDI parameters, there was variability in these relations across white matter regions. Representative scatter plots from white matter regions of the left hemisphere are shown in Fig. 5. We included non-significant age relations as well to illustrate the differential patterns of regional development. From the separation along the ordinate axis one can visualize and infer overall differences between the distinct white matter regions, while slope differences reflect regional variability of development across gestation corrected age.

### Regional Asymmetries

Significant left- and right-hemisphere microstructural comparisons highlighted widespread regional asymmetries (see Table 3). There was a significant (*p* < 0.05, corrected) leftward asymmetry (increased FA, decreased MD, AD, RD) of DTI parameters in the corpus callosum, posterior limb of internal capsule, external capsule, posterior thalamic radiations, sagittal stratum, and inferior fronts-occipital fasciculus. There was a rightward asymmetry in regions of the anterior limb of the internal capsule, cingulum (hippocampal part), stria terminalis, and the superior fronto-occipital fasciculus. NODDI parameters also exhibited patterns regional asymmetries, with leftward asymmetries in the corpus callosum, stria terminalis, and the retrolenticular part of the internal capsule and rightward asymmetries in the superior corona radiata, superior longitudinal fasciculus, and uncinate fasciculus. Age and sex did not relate to asymmetry.

**Table 3:** Left and right hemisphere comparisons in white matter regions.

	FA			MD			AD			RD			νIC			ODI			νISO		
	Left	Right	P	Left	Right	P	Left	Right	P	Left	Right	P	Left	Right	P	Left	Right	P	Left	Right	P
Corpus Callosum	0.33 (0.03)	0.27 (0.02)	<**0.001**	1.17 (0.04)	1.32 (0.09)	<**0.001**	1.66 (0.06)	1.8 (0.12)	<**0.001**	0.96 (0.05)	1.11 (0.08)	<**0.001**	0.23 (0.02)	0.21 (0.02)	<**0.001**	0.07 (0.02)	0.06 (0.02)	<**0.001**	0.09 (0.05)	0.21 (0.09)	<**0.001**
Anterior Limb of Internal Capsule	0.16 (0.02)	0.2 (0.02)	<**0.001**	1.05 (0.09)	1.03 (0.03)	0.050	1.25 (0.09)	1.28 (0.04)	<**0.001**	0.96 (0.08)	0.93 (0.04)	<**0.001**	0.26 (0.05)	0.25 (0.02)	0.3200	0.3 (0.03)	0.23 (0.02)	<**0.001**	0.02 (0.11)	0 (0)	0.0400
Posterior Limb of Internal Capsule	0.37 (0.02)	0.34 (0.02)	<**0.001**	0.92 (0.03)	0.96 (0.03)	<**0.001**	1.31 (0.04)	1.37 (0.04)	<**0.001**	0.72 (0.03)	0.78 (0.03)	<**0.001**	0.32 (0.02)	0.3 (0.02)	<**0.001**	0.15 (0.01)	0.15 (0.02)	0.0500	0 (0.02)	0 (0.01)	0.6700
Retrolenticular Part of Internal Capsule	0.35 (0.02)	0.2 (0.02)	<**0.001**	0.96 (0.03)	1.05 (0.06)	<**0.001**	1.37 (0.05)	1.29 (0.09)	<**0.001**	0.77 (0.03)	0.94 (0.06)	<**0.001**	0.29 (0.02)	0.25 (0.03)	<**0.001**	0.14 (0.02)	0.26 (0.05)	<**0.001**	0 (0.01)	0 (0.02)	0.3100
Anterior Corona Radiata	0.17 (0.03)	0.2 (0.02)	<**0.001**	1.29 (0.11)	1.2 (0.07)	<**0.001**	1.54 (0.11)	1.46 (0.08)	<**0.001**	1.17 (0.11)	1.07 (0.07)	<**0.001**	0.14 (0.09)	0.17 (0.03)	<**0.001**	0.16 (0.07)	0.18 (0.03)	0.0200	0.01 (0.06)	0 (0.02)	0.3900
Superior Corona Radiata	0.2 (0.02)	0.22 (0.02)	<**0.001**	1.17 (0.08)	1.12 (0.06)	<**0.001**	1.43 (0.08)	1.39 (0.07)	<**0.001**	1.05 (0.07)	1 (0.06)	<**0.001**	0.18 (0.03)	0.2 (0.03)	<**0.001**	0.18 (0.03)	0.19 (0.02)	<**0.001**	0 (0.03)	0 (0)	0.3700
Posterior Corona Radiata	0.23 (0.03)	0.21 (0.02)	<**0.001**	1.13 (0.07)	1.12 (0.07)	0.020	1.44 (0.08)	1.4 (0.08)	<**0.001**	1 (0.06)	1 (0.07)	0.910	0.21 (0.03)	0.21 (0.03)	0.6400	0.16 (0.03)	0.19 (0.03)	<**0.001**	0 (0.02)	0 (0)	0.1200
Cingulum (cingular part)	0.14 (0.01)	0.29 (0.02)	<**0.001**	1.14 (0.04)	1.15 (0.05)	<**0.001**	1.31 (0.04)	1.52 (0.07)	<**0.001**	1.05 (0.04)	0.97 (0.05)	<**0.001**	0.2 (0.02)	0.21 (0.02)	<**0.001**	0.3 (0.03)	0.12 (0.03)	<**0.001**	0 (0.03)	0.01 (0.03)	0.0800
Cingulum (hippocampal part)	0.12 (0.01)	0.21 (0.02)	<**0.001**	1.12 (0.03)	1.03 (0.03)	<**0.001**	1.3 (0.04)	1.28 (0.04)	<**0.001**	1.05 (0.03)	0.91 (0.04)	<**0.001**	0.23 (0.02)	0.26 (0.02)	<**0.001**	0.32 (0.03)	0.23 (0.03)	<**0.001**	0.22 (0.16)	0.04 (0.06)	<**0.001**
Stria Terminalis	0.22 (0.02)	0.33 (0.02)	<**0.001**	1.02 (0.04)	1.09 (0.06)	<**0.001**	1.3 (0.05)	1.5 (0.08)	<**0.001**	0.89 (0.04)	0.89 (0.06)	0.590	0.28 (0.02)	0.24 (0.02)	<**0.001**	0.21 (0.02)	0.11 (0.02)	<**0.001**	0.09 (0.09)	0.01 (0.04)	<**0.001**
Superior Longitudinal Fasciculus	0.21 (0.02)	0.16 (0.02)	<**0.001**	1.26 (0.08)	1.22 (0.08)	<**0.001**	1.55 (0.09)	1.44 (0.09)	<**0.001**	1.12 (0.08)	1.12 (0.08)	0.080	0.15 (0.03)	0.17 (0.03)	<**0.001**	0.15 (0.03)	0.23 (0.04)	<**0.001**	0 (0)	0 (0)	0.4200
External Capsule	0.22 (0.01)	0.15 (0.01)	<**0.001**	1.06 (0.04)	1.1 (0.04)	<**0.001**	1.3 (0.04)	1.28 (0.04)	<**0.001**	0.94 (0.04)	1 (0.04)	<**0.001**	0.24 (0.02)	0.22 (0.02)	<**0.001**	0.22 (0.02)	0.31 (0.03)	<**0.001**	0 (0.02)	0 (0.01)	0.8100
Posterior Thalamic Radiation	0.29 (0.02)	0.26 (0.02)	<**0.001**	1.2 (0.06)	1.22 (0.07)	<**0.001**	1.62 (0.08)	1.59 (0.08)	<**0.001**	1.01 (0.06)	1.05 (0.06)	<**0.001**	0.19 (0.02)	0.16 (0.03)	<**0.001**	0.08 (0.03)	0.1 (0.03)	<**0.001**	0.03 (0.06)	0 (0.01)	<**0.001**
Sagittal Stratum	0.26 (0.02)	0.24 (0.02)	<**0.001**	1.2 (0.05)	1.23 (0.06)	<**0.001**	1.54 (0.06)	1.57 (0.07)	<**0.001**	1.04 (0.04)	1.07 (0.06)	<**0.001**	0.18 (0.02)	0.16 (0.03)	<**0.001**	0.12 (0.02)	0.11 (0.03)	0.1400	0.02 (0.04)	0 (0)	<**0.001**
Superior Fronto-Occipital Fasciculus	0.13 (0.02)	0.24 (0.02)	<**0.001**	1.23 (0.12)	1.06 (0.05)	<**0.001**	1.4 (0.13)	1.32 (0.06)	<**0.001**	1.14 (0.11)	0.93 (0.05)	<**0.001**	0.18 (0.1)	0.23 (0.03)	<**0.001**	0.26 (0.06)	0.2 (0.03)	<**0.001**	0.09 (0.17)	0 (0.01)	<**0.001**
Inferior Fronto-Occipital Fasciculus	0.25 (0.02)	0.21 (0.02)	<**0.001**	1.1 (0.04)	1.1 (0.04)	0.320	1.4 (0.05)	1.36 (0.05)	<**0.001**	0.95 (0.04)	0.98 (0.05)	<**0.001**	0.22 (0.02)	0.22 (0.02)	0.2300	0.17 (0.02)	0.22 (0.03)	<**0.001**	0 (0.03)	0 (0.02)	0.9100
Uncinate Fasciculus	0.26 (0.02)	0.21 (0.02)	<**0.001**	1.13 (0.06)	1.1 (0.05)	<**0.001**	1.47 (0.08)	1.36 (0.07)	<**0.001**	0.96 (0.06)	0.98 (0.05)	<**0.001**	0.21 (0.03)	0.23 (0.04)	<**0.001**	0.14 (0.03)	0.21 (0.04)	<**0.001**	0.02 (0.07)	0.13 (0.18)	<**0.001**

Mean and standard deviation of white matter estimates are provided. Significance was defined as p<0.05 (Bonferroni corrected for multiple comparisons). P-values meeting the significance criteria are bolded.

### Sex Differences

Males had higher AD values in the left superior longitudinal fasciculus and right posterior limb of the internal capsule (*p* < 0.05 corrected). These sex differences remained significant after controlling for gestation corrected age.

### Newborn Growth Measures

DTI and NODDI measures were unrelated to head circumference at birth, birth length or birth length after multiple comparison correction.

### Comparison of DTI and NODDI Measures

We observed significant correlations between DTI and NODDI parameters in many white matter tracts (Supplementary Tables 1–4). FA and ν_IC_ were positively correlated in the corpus callosum; anterior and superior corona radiata; posterior thalamic radiations; sagittal stratum, among other right hemispheric white matter regions (Supplementary Table 1). ν_IC_, RD and MD were positively related in all regions except for the left hemisphere anterior limb of the internal capsule and superior fronto-occipital fasciculus (Supplementary Tables 2 and 4), whereas AD was negatively associated with ν_IC_ in all regions except for the left hemisphere superior fronto-occipital fasciculus and right corpus callosum (Supplementary Table 3). DTI parameters and ODI were negatively related in the majority of white matter regions investigated, whereas associations with ν_ISO_ were more infrequent.

## Discussion

We investigated regional characteristics of infant white matter microstructure from a large cohort of one-month old infants using conventional (DTI) and multicompartment (NODDI) models of diffusion. Even within a narrow age range, measures of white matter microstructure varied with gestational age and exhibited extensive left-right differences. Sex differences were minimal and no significant associations emerged between white matter measures and newborn growth markers. Lastly, comparison of DTI and NODDI values demonstrate that although these techniques measure similar aspects of white matter microstructure, they provide differential microstructural information at this early stage of development. These results complement the extant literature on white matter development as well as provide new insights into the neonatal brain, an important step in understanding normative infant brain development not yet influenced by postnatal experiences.

### White Matter Microstructure and Gestational Age Relations

DTI and NODDI parameters varied with gestation corrected age across regions of white matter, indicated by increasing FA, ν_IC_ and ODI and decreasing MD, AD, and RD. These patterns of development are consistent with previous infant studies of smaller sample sizes^17, 20, 21, 43^, and represent the beginning of the nonlinear white matter developmental trajectory reported across childhood^10, 14, 15, 17, 18, 44, 45^ and adolescence and adulthood^26, 46, 47^. DTI diffusivity measures were strongly associated with gestation corrected-age, reflecting broad decreases in total brain water content and increases in membrane density. RD decreases and FA increases within central white matter regions may be indicative of early myelination^10, 48^ or enhanced organization of white matter fibers and bundles^49, 50^.

An advantage of NODDI is that it utilizes biophysical modeling to infer specific attributes of the microstructure. ν_IC_ is interpreted to provide an index of neurite (i.e., axons and dendrites) density, while ODI is representative of the degree of fiber coherence^13^. Hence, our findings with NODDI measures reflect an increasing neurite microstructure, with increases of ν_IC_ corresponding to developing axons and dendrites, while age-relations of ODI are representative of increasing complexity of fiber architecture, such as axonal fanning and bending and dendritic branching.

Regional age variation in white matter microstructure (Fig. 5) suggests that white matter pathways mature at different times and rates. Moreover, this regional variability emphasizes that the central-to-peripheral posterior-to-anterior developmental gradient pattern of white matter development begins as early as one-month. Central white matter regions, such as the corpus callosum, internal capsules, and corona radiata, develop before more peripheral white matter, and posterior tracts develop prior to more anterior ones. This pattern of development is consistent with previous histological^51–53^ and neuroimaging^18, 54–56^ studies of human brain development.

Our findings also indicate that a large proportion of white matter microstructure is established at one-month. Qualitatively, FA and ODI maps (Figs 2–3) at one month appear adult-like, suggesting that the fiber architecture and organization develop *in utero*. On the other hand, developmental processes such as myelination primarily take place postnatally^10, 57^ and are likely to influence subsequent changes in RD and ν_IC_ parameters. Indeed, studies of developing fetuses and preterm newborns find that microstructural anisotropy increases and diffusivity decreases^58–61^, and studies using myelin sensitive measures in children as young as 3 months have demonstrated rapid postnatal myelination^18, 41, 62–64^. Decreased RD and increased ν_IC_ in the internal capsules and corpus callosum suggest that myelin is mainly present in these structures at one month.

### Regional Asymmetries and Sex Differences in White Matter Development

Consistent with previous studies of asymmetry, our results demonstrate lateralization of white microstructural organization of white matter^19^ and myelin content^65^ during infancy. FA asymmetries are congruous to patterns that reflect leftward asymmetry of early developing white matter regions, including the corpus callosum, retrolenticular and posterior portions of the internal capsules, and thalamic radiations; whereas a rightward asymmetry is observed in later developing regions, such as the cingulum and stria terminalis. The exception of this pattern is FA of the uncinate fasciculus. Given the role that developing white matter has on evolving cognitive ability^3, 66^ and that lateralization of cortical function is well established^67^, structural asymmetries of white matter may underlie functional lateralization.

Though previous research finds sexual dimorphism of infant brain volumes^68^, we did not detect widespread white matter microstructure sex differences at one-month of age. Rather, few differences emerged when comparing dMRI indices, with males having larger AD values in the left superior longitudinal fasciculus and right posterior limb of the internal capsule. While our findings are in agreement with similar investigations of developing white matter^41, 54, 69^, others have found male and female differences throughout mid- and later life^26, 70, 71^. This suggests that white matter sexual dimorphism is not apparent at one month, but may emerge at later stages of childhood, adolescence or early adulthood, a time when alterations to concentrations and exposure of sex steroids and other hormones may have an increased effect on brain changes^72^. Still, it is also possible that the sample size of the current study limited our ability to detect dMRI differences between males and females. Thus, comparisons of dMRI metrics from larger cohorts of male and female infants will be critical for identifying possible sex differences during early brain development.

DTI and NODDI parameters correspond to distinct, yet related, models of diffusion. These associations are not surprising, though it is interesting that in some regions DTI and NODDI values are highly correlated, whereas in others, they are not. These patterns of correlations may be partially explained by the neurite density and orientation dispersion that contribute to measures of anisotropy and diffusivity. Alternatively, relations between DTI and NODDI parameters may correspond to regional variations in when specific white matter regions begin to develop. For example, the corpus callosum is an early myelinating region^10^ and FA and ν_IC_ in this region are strongly correlated. In a later maturing tract such as the stria terminalis^10^, FA and ν_IC_ are unrelated. Future studies should continue to incorporate both DTI and NODDI measures to better understand the underlying mechanisms responsible for the associations underlying white matter development.

### Limitations and Conclusions

Our study is not without limitations. First, although DTI metrics reflect changes to microstructure, they are inherently nonspecific and likely reflect a wide variety of neurobiological mechanisms, including changes of myelination, axon diameter and density, or membrane permeability^73–75^. Furthermore, NODDI is a biophysical diffusion model that aims to improve microstructural specificity, yet assumes only three microstructural compartments: intraneurite diffusion, extraneurite diffusion, and isotropic water diffusion. Such an assumption may be an over-simplification of the tissue microstructure, as studies have shown axonal features to vary within a voxel^76–79^. Furthermore, a limitation of our diffusion protocol is that the highest diffusion weighting is 1500 s/mm^2^, while it is recommended to be at least 2000 s/mm^2^. The NODDI model assumes a fixed value for the diffusivity of the intracellular compartment that is suitable for an adult brain^13^. Due to the increased water content of the infant brain, smaller b-values are typically utilized^80^ and therefore we limited the maximum b-value to 1500 s/mm^2^. However, we did adhere to the default value provided in the NODDI MATLAB toolbox^13^ for parameter estimation. Estimated NODDI parameters appear to be appropriate and consistent with values observed by others^14, 16, 17^, however, further investigation of the effects of diffusion-weighting and the default diffusivity are important to investigate in future work.

Birth to two years of age is perhaps the most critical period in which the brain develops. While white matter microstructure undergoes significant change during this time, characterizing the earliest patterns from a normative population are critical to establish a starting point that could be used to examine how postnatal experiences may influence subsequent relationships. To establish such a basis, we investigated measures of white matter microstructure acquired from a large cohort of typically developing one month infants. Even as early as one-month of life, we observe striking biological timing and regional asymmetries beginning to emerge within the developing white matter microstructure. Understanding these early developmental patterns of white matter are essential for appreciating the changes that occur at later stages of growth and informs a deeper understanding of the role of white matter throughout brain maturation.

## Electronic supplementary material

Supplementary Information
