# Supplementary material for: Mapping White Matter Microstructure in the One Month Human Brain
Source: Sci Rep. 2017 Aug 29;7:9759. doi: 10.1038/s41598-017-09915-6 (PMC5575288; doi:10.1038/s41598-017-09915-6)

Mapping White Matter Microstructure in the One Month Human Brain

DC Dean III[1,2], EM Planalp[1,3], W Wooten[2], N Adluru[1], SR Kecskemeti[1], C Frye[2], CK Schmidt[1,2], NL Schmidt[1,2], MA Styner[4,5] HH Goldsmith[1,3], RJ Davidson[1,2,3,6], AL Alexander[1,6,7]

[1]Waisman Center, University of Wisconsin—Madison, Madison, WI, USA
[2]Center for Healthy Minds, University of Wisconsin–Madison, Madison, WI, USA
[3]Department of Psychology, University of Wisconsin—Madison, Madison, Wisconsin, USA.
[4]Department of Psychiatry, University of North Carolina–Chapel Hill, Chapel Hill, NC, USA
[5]Department of Computer Science, University of North Carolina–Chapel Hill, Chapel Hill, NC, USA
[6]Department of Psychiatry, University of Wisconsin—Madison School of Medicine and Public Health, Madison, WI, USA
[7]Department of Medical Physics, University of Wisconsin—Madison School of Medicine and Public Health, Madison, WI, USA

Address Correspondence to: Douglas C. Dean III
Waisman Center
University of Wisconsin-Madison
Madison, WI, USA, 53705
Tel. # +1 608.263.6318
Email: deaniii@wisc.edu

Running Title: White matter microstructure in infants.

Manuscript prepared for submission to Scientific Reports (Article)

Number of Supplemental Tables: 4
Number of Supplemental Figures: 1

Supplementary Table 1: Correlations between FA and NODDI model estimates. Significant correlations ($p<0.05$, Bonferroni corrected) are bolded.

	ν_{IC}			ODI			ν_{ISO}		
**Left Hemisphere**	R	T-statistic	P-Value	R	T-statistic	P-Value	R	T-statistic	P-Value
Corpus Callosum	0.5049	5.9072	**<0.000001**	-0.4818	-5.5535	**<0.000001**	0.2237	2.3182	0.022435
Anterior Limb of Internal Capsule	0.0723	0.7317	0.466015	-0.6099	-7.7722	**<0.000001**	-0.1704	-1.7467	0.083700
Posterior Limb of Internal Capsule	0.4016	4.4288	**<0.000001**	-0.6200	-7.9810	**<0.000001**	0.2509	2.6178	0.010196
Retrolenticular Part of Internal Capsule	0.2705	2.8381	0.005476	-0.5163	-6.0879	**<0.000001**	0.3988	4.3920	**<0.000001**
Anterior Corona Radiata	0.6025	7.6234	**<0.000001**	0.0055	0.0559	0.955529	-0.6927	-9.6988	**<0.000001**
Superior Corona Radiata	0.6052	7.6770	**<0.000001**	0.1086	1.1028	0.272691	-0.4368	-4.9039	**<0.000001**
Posterior Corona Radiata	0.3583	3.8755	0.000188	-0.2901	-3.0612	0.002818	-0.0632	-0.6400	0.523626
Cingulum (cingular part)	0.2483	2.5885	0.011046	-0.4032	-4.4497	**<0.000001**	0.1020	1.0359	0.302714
Cingulum (hippocampal part)	0.2673	2.8020	0.006078	-0.4975	-5.7922	**<0.000001**	0.0467	0.4723	0.637705
Stria Terminalis	0.0710	0.7186	0.474024	-0.4680	-5.3479	**<0.000001**	0.0041	0.0410	0.967403
Superior Longitudinal Fasciculus	0.7485	11.3989	**<0.000001**	0.3383	3.6305	0.000444	-0.6012	-7.5987	**<0.000001**
External Capsule	0.3012	3.1902	0.001890	-0.3688	-4.0069	0.000117	-0.0660	-0.6680	0.505656
Posterior Thalamic Radiation	0.5621	6.8641	**<0.000001**	-0.1321	-1.3456	0.181410	-0.0686	-0.6949	0.488687
Sagittal Stratum	0.5371	6.4308	**<0.000001**	-0.1843	-1.8934	0.061134	0.0153	0.1548	0.877298
Superior Fronto-Occipital Fasciculus	-0.1257	-1.2792	0.203744	-0.6140	-7.8556	**<0.000001**	0.2548	2.6615	0.009039
Inferior Fronto-Occipital Fasciculus	0.1804	1.8526	0.066833	-0.4857	-5.6123	**<0.000001**	0.2611	2.7315	0.007430
Uncinate Fasciculus	0.0661	0.6689	0.505059	-0.4389	-4.9330	**<0.000001**	0.2600	2.7193	0.007691
**Right Hemisphere**									
Corpus Callosum	0.4481	5.0623	**<0.000001**	-0.0838	-0.8492	0.397748	-0.1922	-1.9782	0.050608
Anterior Limb of Internal Capsule	0.2402	2.4990	0.014052	-0.6359	-8.3206	**<0.000001**	0.0791	0.8016	0.424628
Posterior Limb of Internal Capsule	0.2876	3.0332	0.003069	-0.5386	-6.4564	**<0.000001**	0.0800	0.8105	0.419548
Retrolenticular Part of Internal Capsule	-0.0474	-0.4795	0.632607	-0.5052	-5.9124	**<0.000001**	0.3070	3.2580	0.001524
Anterior Corona Radiata	0.5876	7.3346	**<0.000001**	0.0918	0.9309	0.354100	-0.4959	-5.7676	**<0.000001**
Superior Corona Radiata	0.6546	8.7462	**<0.000001**	0.0892	0.9042	0.368015	-0.4827	-5.5672	**<0.000001**
Posterior Corona Radiata	0.5863	7.3098	**<0.000001**	0.0478	0.4831	0.630070	-0.3980	-4.3810	**<0.000001**
Cingulum (cingular part)	0.4755	5.4588	**<0.000001**	-0.2846	-2.9988	0.003406	0.1479	1.5102	0.134075
Cingulum (hippocampal part)	0.4208	4.6853	**<0.000001**	-0.6574	-8.8115	**<0.000001**	0.2833	2.9834	0.003567
Stria Terminalis	0.5465	6.5911	**<0.000001**	-0.2165	-2.2399	0.027267	-0.1529	-1.5629	0.121178
Superior Longitudinal Fasciculus	0.3655	3.9655	0.000136	-0.1580	-1.6158	0.109227	-0.2097	-2.1657	0.032666
External Capsule	0.4449	5.0174	**<0.000001**	-0.3304	-3.5358	0.000613	-0.2210	-2.2886	0.024166
Posterior Thalamic Radiation	0.6139	7.8543	**<0.000001**	0.1537	1.5708	0.119335	-0.3261	-3.4839	0.000730
Sagittal Stratum	0.5638	6.8941	**<0.000001**	0.0955	0.9691	0.334773	-0.3662	-3.9749	0.000132
Superior Fronto-Occipital Fasciculus	0.3447	3.7092	0.000339	-0.5203	-6.1538	**<0.000001**	0.1150	1.1688	0.245193
Inferior Fronto-Occipital Fasciculus	0.4559	5.1730	**<0.000001**	-0.5566	-6.7668	**<0.000001**	-0.2142	-2.2146	0.029014
Uncinate Fasciculus	0.0170	0.1717	0.864013	-0.4681	-5.3499	**<0.000001**	0.1594	1.6312	0.105942

Supplementary Table 2: Correlations between MD and NODDI model estimates. Significant correlations ($p<0.05$, Bonferroni corrected) are bolded.

	ν_{IC}			ODI			ν_{ISO}		
**Left Hemisphere**	R	T-statistic	P-Value	R	T-statistic	P-Value	R	T-statistic	P-Value
Corpus Callosum	-0.7023	-9.9629	**<0.000001**	-0.3961	-4.3564	**0.00003**	-0.3658	-3.9699	0.00013
Anterior Limb of Internal Capsule	0.3738	4.0701	0.00009	-0.2734	-2.8703	0.00499	0.6894	9.6126	**<0.000001**
Posterior Limb of Internal Capsule	-0.8225	-14.6042	**<0.000001**	-0.3528	-3.8083	0.00024	-0.1876	-1.9289	0.05652
Retrolenticular Part of Internal Capsule	-0.7860	-12.8412	**<0.000001**	-0.4525	-5.1247	**<0.000001**	0.0292	0.2954	0.76830
Anterior Corona Radiata	-0.6651	-8.9946	**<0.000001**	-0.1722	-1.7660	0.08039	-0.2894	-3.0537	0.00288
Superior Corona Radiata	-0.9118	-22.4315	**<0.000001**	-0.7732	-12.3134	**<0.000001**	-0.0712	-0.7206	0.47281
Posterior Corona Radiata	-0.8416	-15.7397	**<0.000001**	-0.6652	-8.9981	**<0.000001**	0.1154	1.1730	0.24353
Cingulum (cingular part)	-0.8680	-17.6531	**<0.000001**	-0.6682	-9.0712	**<0.000001**	-0.1625	-1.6628	0.09943
Cingulum (hippocampal part)	-0.6480	-8.5934	**<0.000001**	-0.4714	-5.3988	**<0.000001**	-0.0599	-0.6058	0.54599
Stria Terminalis	-0.5406	-6.4892	**<0.000001**	-0.2695	-2.8267	0.00566	0.4149	4.6049	0.00001
Superior Longitudinal Fasciculus	-0.9396	-27.7185	**<0.000001**	-0.7579	-11.7336	**<0.000001**	0.2541	2.6534	0.00925
External Capsule	-0.8600	-17.0199	**<0.000001**	-0.5319	-6.3436	**<0.000001**	-0.3537	-3.8186	0.00023
Posterior Thalamic Radiation	-0.7313	-10.8273	**<0.000001**	-0.7241	-10.6024	**<0.000001**	0.2835	2.9852	0.00355
Sagittal Stratum	-0.7906	-13.0409	**<0.000001**	-0.7004	-9.9098	**<0.000001**	-0.0333	-0.3365	0.73716
Superior Fronto-Occipital Fasciculus	0.2136	2.2084	0.0295	-0.5882	-7.3453	**<0.000001**	0.4073	4.5043	0.00002
Inferior Fronto-Occipital Fasciculus	-0.8481	-16.1686	**<0.000001**	-0.6303	-8.1985	**<0.000001**	-0.4468	-5.0445	**<0.000001**
Uncinate Fasciculus	-0.7062	-10.0727	**<0.000001**	-0.5703	-7.0124	**<0.000001**	-0.4000	-4.4074	0.00003
**Right Hemisphere**									
Corpus Callosum	-0.4336	-4.8597	**<0.000001**	-0.5966	-7.5088	**<0.000001**	0.3743	4.0765	0.00009
Anterior Limb of Internal Capsule	-0.7544	-11.6068	**<0.000001**	-0.2253	-2.3350	0.02150	NA	NA	NA
Posterior Limb of Internal Capsule	-0.8248	-14.7297	**<0.000001**	-0.2942	-3.1092	0.00243	-0.2769	-2.9104	0.00443
Retrolenticular Part of Internal Capsule	-0.9242	-24.4475	**<0.000001**	-0.6988	-9.8656	**<0.000001**	-0.1306	-1.3300	0.18649
Anterior Corona Radiata	-0.8768	-18.4195	**<0.000001**	-0.6300	-8.1938	**<0.000001**	-0.0995	-1.0097	0.31501
Superior Corona Radiata	-0.9213	-23.9325	**<0.000001**	-0.6674	-9.0505	**<0.000001**	-0.1483	-1.5145	0.13299
Posterior Corona Radiata	-0.9443	-28.9668	**<0.000001**	-0.6067	-7.7077	**<0.000001**	0.3887	4.2605	0.00005
Cingulum (cingular part)	-0.8615	-17.1386	**<0.000001**	-0.6096	-7.7660	**<0.000001**	-0.0460	-0.4648	0.64308
Cingulum (hippocampal part)	-0.7722	-12.2745	**<0.000001**	-0.1248	-1.2705	0.20681	-0.2556	-2.6697	0.00883
Stria Terminalis	-0.7712	-12.2333	**<0.000001**	-0.5463	-6.5878	**<0.000001**	0.4747	5.4470	**<0.000001**
Superior Longitudinal Fasciculus	-0.9515	-31.2362	**<0.000001**	-0.7296	-10.7752	**<0.000001**	-0.0644	-0.6513	0.51632
External Capsule	-0.8084	-13.8695	**<0.000001**	-0.4167	-4.6293	**0.00001**	-0.3096	-3.2881	0.00138
Posterior Thalamic Radiation	-0.9359	-26.8278	**<0.000001**	-0.7852	-12.8073	**<0.000001**	0.1710	1.7533	0.08255
Sagittal Stratum	-0.9161	-23.0790	**<0.000001**	-0.7423	-11.1895	**<0.000001**	0.3966	4.3636	0.00003
Superior Fronto-Occipital Fasciculus	-0.8976	-20.5598	**<0.000001**	-0.4905	-5.6843	**<0.000001**	-0.1331	-1.3566	0.17791
Inferior Fronto-Occipital Fasciculus	-0.7266	-10.6805	**<0.000001**	-0.0131	-0.1322	0.89509	-0.1977	-2.0369	0.04425
Uncinate Fasciculus	-0.6469	-8.5671	**<0.000001**	-0.5061	-5.9265	**<0.000001**	-0.3168	-3.3734	0.00105

Supplementary Table 3: Correlations between AD and NODDI model estimates. Significant correlations ($p<0.05$, Bonferroni corrected) are bolded.

	ν_{IC}			ODI			ν_{ISO}		
**Left Hemisphere**	R	T-statistic	P-Value	R	T-statistic	P-Value	R	T-statistic	P-Value
Corpus Callosum	-0.4250	-4.7418	**0.0000**	-0.8754	-18.2895	**0.0000**	-0.3818	-4.1720	**0.0001**
Anterior Limb of Internal Capsule	0.3974	4.3734	**0.0000**	-0.4256	-4.7503	0.0050	0.7006	9.9175	**<0.000001**
Posterior Limb of Internal Capsule	-0.5447	-6.5590	**<0.000001**	-0.7574	-11.7168	0.0002	-0.0024	-0.0242	0.9807
Retrolenticular Part of Internal Capsule	-0.4876	-5.6407	**<0.000001**	-0.8332	-15.2162	**0.0000**	0.0054	0.0541	0.9569
Anterior Corona Radiata	-0.6645	-8.9805	**<0.000001**	-0.2428	-2.5280	0.0804	-0.3102	-3.2949	0.0014
Superior Corona Radiata	-0.8480	-16.1560	**<0.000001**	-0.8750	-18.2572	**<0.000001**	-0.0598	-0.6046	0.5468
Posterior Corona Radiata	-0.7456	-11.2986	**<0.000001**	-0.8469	-16.0825	**<0.000001**	0.0826	0.8373	0.4044
Cingulum (cingular part)	-0.7962	-13.2922	**<0.000001**	-0.7989	-13.4148	**<0.000001**	-0.1879	-1.9326	0.0561
Cingulum (hippocampal part)	-0.5456	-6.5745	**<0.000001**	-0.6664	-9.0263	**<0.000001**	-0.0614	-0.6213	0.5358
Stria Terminalis	-0.4513	-5.1081	**0.0000**	-0.5743	-7.0847	0.0057	0.3984	4.3862	**0.0000**
Superior Longitudinal Fasciculus	-0.8560	-16.7232	**<0.000001**	-0.8708	-17.8896	**<0.000001**	0.2416	2.5150	0.0135
External Capsule	-0.7208	-10.5019	**<0.000001**	-0.7504	-11.4674	**<0.000001**	-0.2738	-2.8755	0.0049
Posterior Thalamic Radiation	-0.5444	-6.5549	**<0.000001**	-0.8980	-20.6075	**<0.000001**	0.4277	4.7791	**0.0000**
Sagittal Stratum	-0.5282	-6.2824	**<0.000001**	-0.8908	-19.7991	**<0.000001**	0.1351	1.3772	0.1715
Superior Fronto-Occipital Fasciculus	0.1747	1.7919	0.0761	-0.7050	-10.0405	**<0.000001**	0.4007	4.4168	**0.0000**
Inferior Fronto-Occipital Fasciculus	-0.7368	-11.0057	**<0.000001**	-0.8968	-20.4705	**<0.000001**	-0.4958	-5.7653	**<0.000001**
Uncinate Fasciculus	-0.6235	-8.0545	**<0.000001**	-0.8100	-13.9486	**<0.000001**	-0.4698	-5.3745	**<0.000001**
**Right Hemisphere**									
Corpus Callosum	-0.2823	-2.9725	0.0037	-0.7219	-10.5347	**<0.000001**	0.4287	4.7918	**0.0000**
Anterior Limb of Internal Capsule	-0.6985	-9.8582	**<0.000001**	-0.5842	-7.2702	0.0215	NA	NA	NA
Posterior Limb of Internal Capsule	-0.7036	-10.0001	**<0.000001**	-0.7270	-10.6923	0.0024	-0.2372	-2.4661	0.0153
Retrolenticular Part of Internal Capsule	-0.8751	-18.2653	**<0.000001**	-0.8676	-17.6227	**<0.000001**	-0.1053	-1.0696	0.2873
Anterior Corona Radiata	-0.8528	-16.4887	**<0.000001**	-0.7916	-13.0822	**<0.000001**	-0.0803	-0.8132	0.4180
Superior Corona Radiata	-0.8945	-20.2044	**<0.000001**	-0.8111	-14.0050	**<0.000001**	-0.1384	-1.4112	0.1612
Posterior Corona Radiata	-0.8946	-20.2150	**<0.000001**	-0.7715	-12.2465	**<0.000001**	0.3277	3.5028	0.0007
Cingulum (cingular part)	-0.5802	-7.1938	**<0.000001**	-0.8777	-18.4957	**<0.000001**	-0.0856	-0.8673	0.3878
Cingulum (hippocampal part)	-0.4184	-4.6523	**0.0000**	-0.6700	-9.1147	0.2068	-0.0652	-0.6599	0.5108
Stria Terminalis	-0.5939	-7.4558	**<0.000001**	-0.8308	-15.0775	**<0.000001**	0.4914	5.6978	**<0.000001**
Superior Longitudinal Fasciculus	-0.9138	-22.7288	**<0.000001**	-0.8177	-14.3477	**<0.000001**	-0.0578	-0.5845	0.5602
External Capsule	-0.7530	-11.5555	**<0.000001**	-0.6378	-8.3643	**0.0000**	-0.3390	-3.6391	0.0004
Posterior Thalamic Radiation	-0.8434	-15.8536	**<0.000001**	-0.9252	-24.6156	**<0.000001**	0.1782	1.8288	0.0704
Sagittal Stratum	-0.8732	-18.0923	**<0.000001**	-0.8679	-17.6458	**<0.000001**	0.3600	3.8976	0.0002
Superior Fronto-Occipital Fasciculus	-0.7460	-11.3133	**<0.000001**	-0.7957	-13.2686	**<0.000001**	-0.1506	-1.5388	0.1270
Inferior Fronto-Occipital Fasciculus	-0.6162	-7.9009	**<0.000001**	-0.4089	-4.5250	0.8951	-0.2746	-2.8837	0.0048
Uncinate Fasciculus	-0.6075	-7.7249	**<0.000001**	-0.7181	-10.4222	**<0.000001**	-0.3913	-4.2949	**0.0000**

Supplementary Table 4: Correlations between RD and NODDI model estimates. Significant correlations ($p<0.05$, Bonferroni corrected) are bolded.

	ν_{IC}			ODI			ν_{ISO}		
**Left Hemisphere**	R	T-statistic	P-Value	R	T-statistic	P-Value	R	T-statistic	P-Value
Corpus Callosum	0.5049	5.9072	**<0.000001**	-0.4818	-5.5535	**<0.000001**	0.2237	2.3182	0.022435
Anterior Limb of Internal Capsule	0.0723	0.7317	0.466015	-0.6099	-7.7722	**<0.000001**	-0.1704	-1.7467	0.083700
Posterior Limb of Internal Capsule	0.4016	4.4288	**<0.000001**	-0.6200	-7.9810	**<0.000001**	0.2509	2.6178	0.010196
Retrolenticular Part of Internal Capsule	0.2705	2.8381	0.005476	-0.5163	-6.0879	**<0.000001**	0.3988	4.3920	**<0.000001**
Anterior Corona Radiata	0.6025	7.6234	**<0.000001**	0.0055	0.0559	0.955529	-0.6927	-9.6988	**<0.000001**
Superior Corona Radiata	0.6052	7.6770	**<0.000001**	0.1086	1.1028	0.272691	-0.4368	-4.9039	**<0.000001**
Posterior Corona Radiata	0.3583	3.8755	0.000188	-0.2901	-3.0612	0.002818	-0.0632	-0.6400	0.523626
Cingulum (cingular part)	0.2483	2.5885	0.011046	-0.4032	-4.4497	**<0.000001**	0.1020	1.0359	0.302714
Cingulum (hippocampal part)	0.2673	2.8020	0.006078	-0.4975	-5.7922	**<0.000001**	0.0467	0.4723	0.637705
Stria Terminalis	0.0710	0.7186	0.474024	-0.4680	-5.3479	**<0.000001**	0.0041	0.0410	0.967403
Superior Longitudinal Fasciculus	0.7485	11.3989	**<0.000001**	0.3383	3.6305	0.000444	-0.6012	-7.5987	**<0.000001**
External Capsule	0.3012	3.1902	0.001890	-0.3688	-4.0069	0.000117	-0.0660	-0.6680	0.505656
Posterior Thalamic Radiation	0.5621	6.8641	**<0.000001**	-0.1321	-1.3456	0.181410	-0.0686	-0.6949	0.488687
Sagittal Stratum	0.5371	6.4308	**<0.000001**	-0.1843	-1.8934	0.061134	0.0153	0.1548	0.877298
Superior Fronto-Occipital Fasciculus	-0.1257	-1.2792	0.203744	-0.6140	-7.8556	**<0.000001**	0.2548	2.6615	0.009039
Inferior Fronto-Occipital Fasciculus	0.1804	1.8526	0.066833	-0.4857	-5.6123	**<0.000001**	0.2611	2.7315	0.007430
Uncinate Fasciculus	0.0661	0.6689	0.505059	-0.4389	-4.9330	**<0.000001**	0.2600	2.7193	0.007691
**Right Hemisphere**									
Corpus Callosum	0.4481	5.0623	**<0.000001**	-0.0838	-0.8492	0.397748	-0.1922	-1.9782	0.050608
Anterior Limb of Internal Capsule	0.2402	2.4990	0.014052	-0.6359	-8.3206	**<0.000001**	0.0791	0.8016	0.424628
Posterior Limb of Internal Capsule	0.2876	3.0332	0.003069	-0.5386	-6.4564	**<0.000001**	0.0800	0.8105	0.419548
Retrolenticular Part of Internal Capsule	-0.0474	-0.4795	0.632607	-0.5052	-5.9124	**<0.000001**	0.3070	3.2580	0.001524
Anterior Corona Radiata	0.5876	7.3346	**<0.000001**	0.0918	0.9309	0.354100	-0.4959	-5.7676	**<0.000001**
Superior Corona Radiata	0.6546	8.7462	**<0.000001**	0.0892	0.9042	0.368015	-0.4827	-5.5672	**<0.000001**
Posterior Corona Radiata	0.5863	7.3098	**<0.000001**	0.0478	0.4831	0.630070	-0.3980	-4.3810	**<0.000001**
Cingulum (cingular part)	0.4755	5.4588	**<0.000001**	-0.2846	-2.9988	0.003406	0.1479	1.5102	0.134075
Cingulum (hippocampal part)	0.4208	4.6853	**<0.000001**	-0.6574	-8.8115	**<0.000001**	0.2833	2.9834	0.003567
Stria Terminalis	0.5465	6.5911	**<0.000001**	-0.2165	-2.2399	0.027267	-0.1529	-1.5629	0.121178
Superior Longitudinal Fasciculus	0.3655	3.9655	0.000136	-0.1580	-1.6158	0.109227	-0.2097	-2.1657	0.032666
External Capsule	0.4449	5.0174	**<0.000001**	-0.3304	-3.5358	0.000613	-0.2210	-2.2886	0.024166
Posterior Thalamic Radiation	0.6139	7.8543	**<0.000001**	0.1537	1.5708	0.119335	-0.3261	-3.4839	0.000730
Sagittal Stratum	0.5638	6.8941	**<0.000001**	0.0955	0.9691	0.334773	-0.3662	-3.9749	0.000132
Superior Fronto-Occipital Fasciculus	0.3447	3.7092	0.000339	-0.5203	-6.1538	**<0.000001**	0.1150	1.1688	0.245193
Inferior Fronto-Occipital Fasciculus	0.4559	5.1730	**<0.000001**	-0.5566	-6.7668	**<0.000001**	-0.2142	-2.2146	0.029014
Uncinate Fasciculus	0.0170	0.1717	0.864013	-0.4681	-5.3499	**<0.000001**	0.1594	1.6312	0.105942

Supplementary Figure Captions

Supplementary Figure 1: Examined white matter region template priors overlaid on the population averaged FA map. Note, while left and right hemispheres are not depicted, template priors were separated by hemisphere.

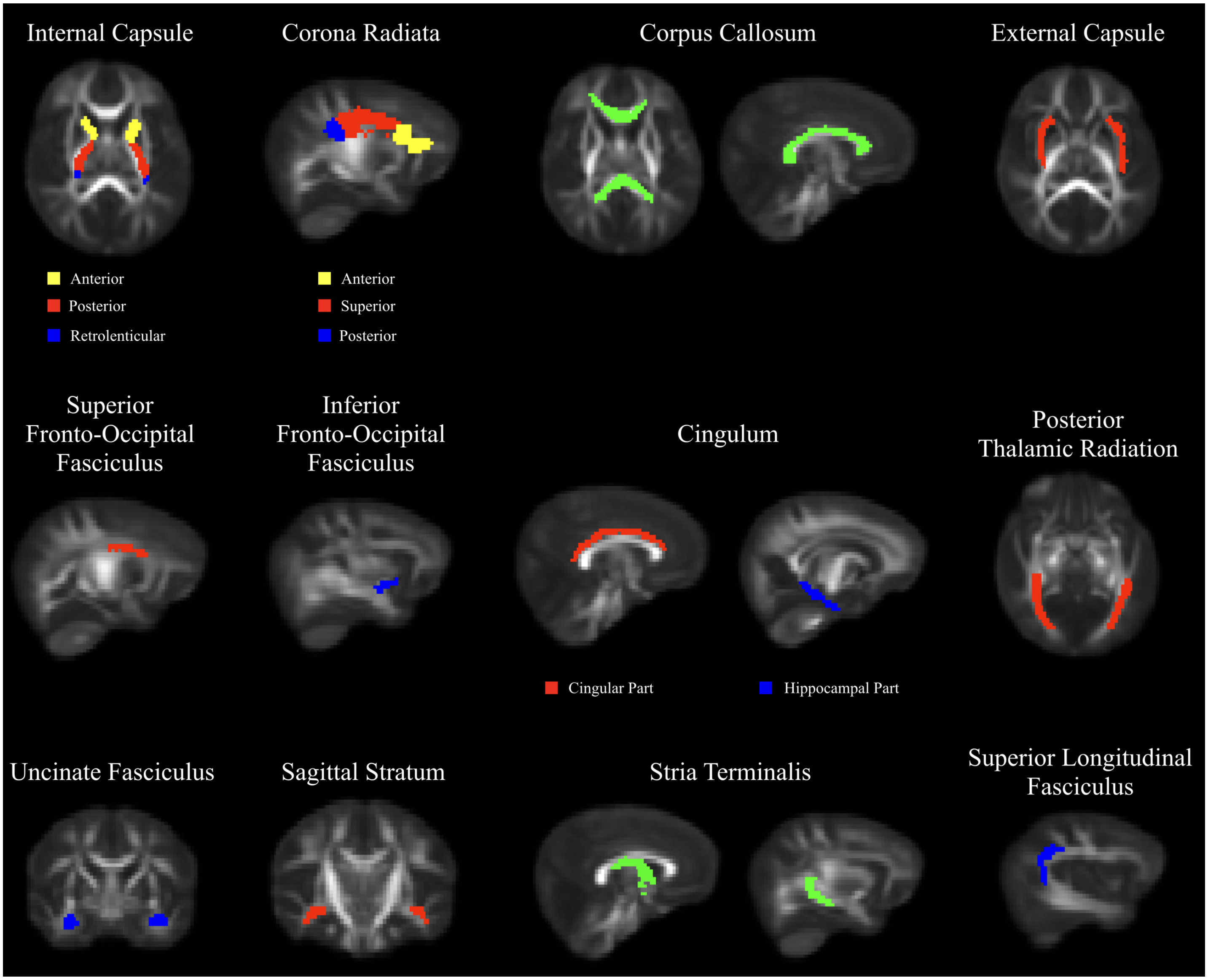

Supplement: Supplementary file 1 — Supplementary Information [file 41598_2017_9915_MOESM1_ESM.pdf]
